# Pediatric Transverse Myelitis: A Case Report

**DOI:** 10.7759/cureus.117305

**Published:** 2026-10-01

**Authors:** Kelly Fong, Daniel Rossie

**Affiliations:** 1 Emergency Medicine, Loma Linda University Medical Center, Loma Linda, USA

**Keywords:** case report, emergency medicine, neurology, pediatrics, transverse myelitis

## Abstract

Transverse myelitis (TM) is a rare inflammatory demyelinating disorder affecting the spinal cord. Although uncommon in children, approximately 20% of cases occur in the pediatric population. Children tend to have a better prognosis than adults. The etiology of TM is broad and includes, but is not limited to, viral and bacterial infections, association with other neurological disorders, and post-vaccination events. This case report describes a 16-year-old male patient who developed acute neurological deficits and urinary retention over several days. The patient was initially evaluated in the emergency department of a county hospital and was subsequently transferred to a tertiary pediatric hospital for further management. An extensive workup, including laboratory studies and imaging, revealed findings concerning for TM. The patient’s symptoms did not resolve following a course of steroids. Therefore, intravenous immunoglobulin (IVIG) was added on the recommendation of the pediatric neuroimmunology team, resulting in resolution of his symptoms. He was eventually discharged home with full recovery of function and mobility. This case highlights a neurological presentation that can mimic other disorders and requires an extensive diagnostic workup for definitive diagnosis. Awareness of TM is important for early recognition and treatment, particularly in the emergency department, given the potential for rapid progression and significant morbidity.

## Introduction

Transverse myelitis (TM) is a relatively rare inflammatory demyelinating disorder that can affect individuals of any age. It involves inflammation across a segment of the spinal cord and may present with numbness and weakness that can progress over hours to weeks. Patients may also present with neck or back pain, neurological deficits, and bladder or bowel dysfunction. TM is more common in adults but occurs in approximately 20% of cases in children [1]. Children generally have better outcomes than adults, with approximately half achieving complete recovery within two years [1]. TM can present similarly to other neurological disorders, such as Guillain-Barré syndrome. It may occur as a post-infectious complication, including following viral or bacterial infections or vaccination, or may be associated with other neurological disorders, including multiple sclerosis, acute disseminated encephalomyelitis, myelin oligodendrocyte glycoprotein antibody-associated disease, and neuromyelitis optica spectrum disorder, in both adults and children. The presence of TM may indicate an underlying neurological disease, although up to 60% of cases may be idiopathic [2]. The nadir of symptoms is usually reached more than four hours but within 21 days of symptom onset [3]. In this case, we report a 16-year-old male patient who presented to the emergency department with urinary retention and acute bilateral lower-extremity weakness and numbness and was subsequently diagnosed with TM during his hospital course. The patient experienced rapid progression of neurological deficits. Given that early recognition and treatment of TM may improve outcomes, TM should be considered in the differential diagnosis of patients presenting with acute neurological deficits in the emergency department.

## Case presentation

A 16-year-old male patient with a history of dyslipidemia initially presented to the county hospital emergency department via ambulance with urinary retention and bilateral lower-extremity numbness and weakness of two days’ duration. The patient reported that the numbness began in his feet and progressed to his lower abdomen. He had previously been evaluated at another community hospital for urinary retention, where he underwent straight catheterization and was subsequently discharged. Because the urinary retention persisted, he returned for reevaluation at the county hospital. Approximately four days before presentation, the patient had been evaluated at another community emergency department for symptoms of pain between his eyes and purulent nasal discharge and was diagnosed with suspected bacterial sinusitis. He was discharged with antibiotics. Urinary retention subsequently developed on the fourth day after starting the antibiotics, prompting evaluation at an outside hospital, where straight catheterization was performed once before he was discharged. The patient discontinued the antibiotics because he was concerned that the medication might be causing the urinary retention. He denied any other symptoms at that time. 

On assessment, physical examination revealed decreased sensation and weakness in both lower extremities, with a sensory level extending to approximately T6. Sensation was intact above the T6 level. Motor strength was 5/5 in both upper extremities and 1/5 in both lower extremities, and the patient was unable to lift either leg against gravity. Clonus was absent, as were reflexes in both lower extremities. Babinski signs were absent bilaterally. The patient was alert and oriented, with a Glasgow Coma Scale score of 15, and cranial nerve examination (III-XII) was intact.

The initial emergency department workup included a complete blood count (CBC), comprehensive metabolic panel, inflammatory markers including erythrocyte sedimentation rate (ESR) and C-reactive protein (CRP), cerebrospinal fluid (CSF) studies and culture, urine studies, and magnetic resonance imaging (MRI) of the brain and spine. A lumbar puncture was performed to obtain CSF samples. Initial laboratory investigations revealed leukocytosis on CBC, elevated ESR and CRP, and absent oligoclonal bands. CSF analysis demonstrated pleocytosis and elevated protein, while CSF glucose was within normal limits. CSF culture and the meningitis/encephalitis panel were negative. These findings, including CSF pleocytosis and elevated protein, were concerning for spinal cord inflammation, while the normal CSF glucose and negative CSF culture and meningitis/encephalitis panel made bacterial meningitis less likely. The Monospot test was positive, whereas the respiratory viral panel was negative (Table 1). Additional investigations for immune-mediated causes of transverse myelitis were not obtained during the initial emergency department evaluation before transfer to the tertiary care center.

**Table 1:** Laboratory findings WBC: white blood cells, CSF: cerebrospinal fluid.

Lab tests	Value	Reference range and units
WBC	15.7	4.3-11.3 x 10^9^/L
Sedimentation rate, Westergren	78	0-15 mm/hr
C-reactive protein	6.86	0-0.30 mg/dL
Oligoclonal banding	Absent	Absent
Monospot test	Positive	-
Protein, CSF	121	15-45 mg/dL
Glucose, CSF	62	40-70 mg/dL
CSF panel		
White blood cells, CSF	186	0-5/mm^3^
Red blood cells, CSF	159	<1/mm^3^
Mono%, CSF	98	94%-00%
Neut%, CSF	2	0%-6%
Clarity/Color CSF	Slightly hazy, colorless	-
Culture, CSF		
Gram stain	1+ WBC, no organisms seen. No growth in 72 hours	-
Meningitis/encephalitis panel	Negative	-
Respiratory viral panel	Negative	-

The patient developed a fever while in the emergency department, with a temperature of 102.8°F. A Foley catheter was placed for urinary retention. Neurosurgery was consulted after MRI revealed concern for a possible diffuse spinal cord infarction versus long-segment transverse myelitis, with imaging demonstrating T2 hyperintensity extending caudally from the C2-C3 level along the length of the spinal cord (Figure 1). The neurosurgery team at the county hospital recommended transfer to a tertiary care children’s hospital and admission to the pediatric intensive care unit for close monitoring of the patient’s neurological status, particularly for progression of weakness. 

**Figure 1:**
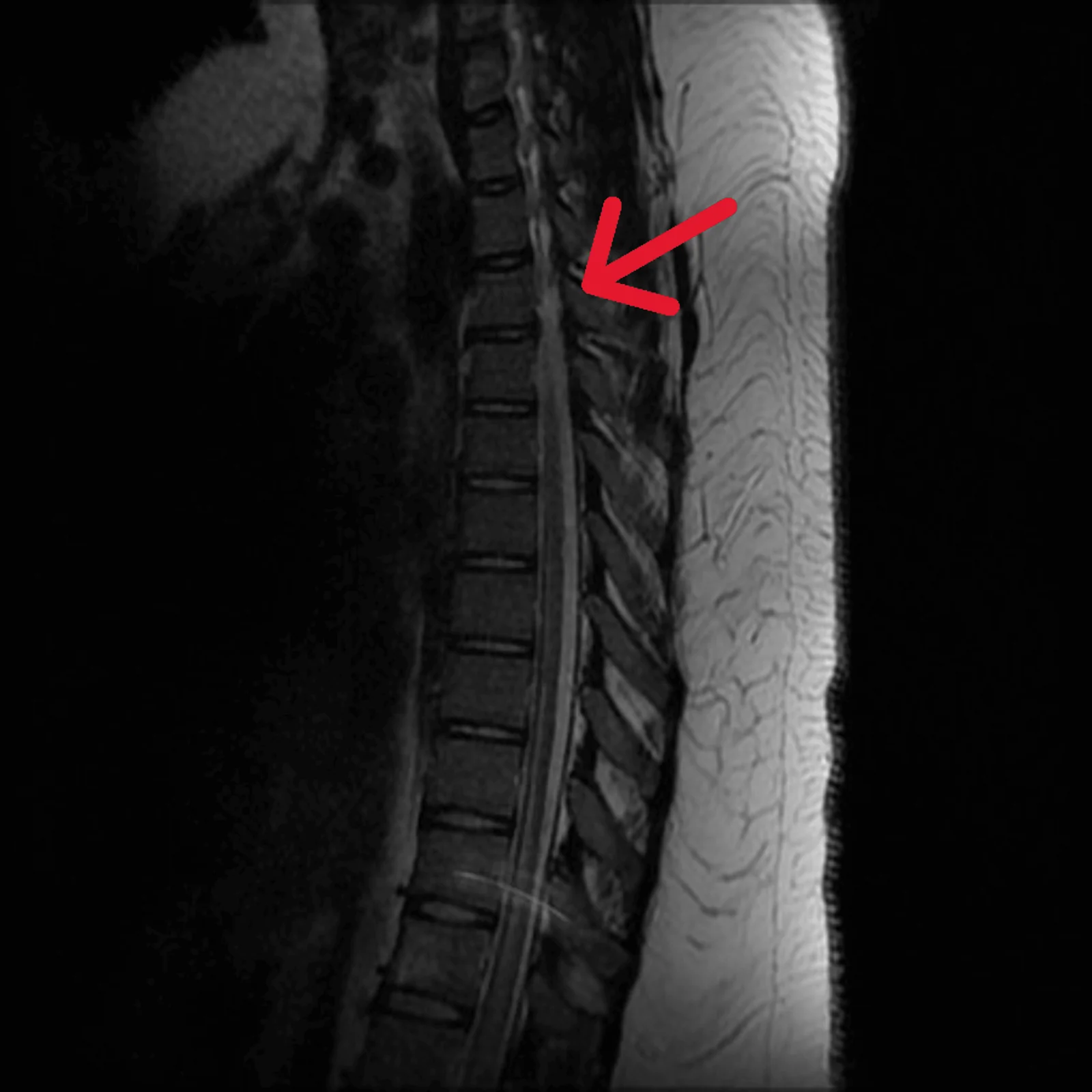
Magnetic resonance imaging of the thoracic spine concerning for diffuse cord infarct versus long-segment transverse myelitis with T2 hyperintensity starting from C2-C3 caudally the length of the spinal cord

At the tertiary children’s hospital, pediatric neurosurgery was consulted and recommended repeat MRI of the spine with and without contrast, as well as consultations with pediatric neurology and infectious disease. The infectious disease team recommended continuing empiric treatment for meningitis while awaiting further infectious workup. The patient received a dose of dexamethasone before evaluation by neurology, with subsequent improvement in sensation and proprioception. Neurology reviewed the imaging and laboratory studies and suspected TM based on the presence of CSF pleocytosis and elevated protein, with negative CSF cultures, findings concerning for spinal cord inflammation and less suggestive of bacterial meningitis. MRI also demonstrated T2 hyperintensity extending caudally from the C2-C3 level along the length of the spinal cord. Neurology recommended continuing corticosteroid therapy and adding IVIG. During the hospital course, the patient’s inflammatory markers decreased, and CSF cultures remained negative. Antibiotics were discontinued based on the infectious disease specialist’s recommendations, as a bacterial etiology was considered less likely. The patient completed a four-day course of IVIG and received intravenous corticosteroids for five days before transitioning to oral steroids. His symptoms improved during the hospital course, with return of movement in both lower extremities. At discharge, the patient was able to void spontaneously and ambulate without difficulty. The Foley catheter was therefore removed. The patient was discharged on a tapered course of prednisone.

## Discussion

TM is a rare disorder, particularly in the pediatric population. Patients presenting with neurological signs of spinal cord dysfunction should have TM included in the differential diagnosis, as early recognition and treatment may contribute to faster recovery and reduced morbidity. In the present case, the patient demonstrated significant improvement after treatment with steroids and IVIG and returned to his clinical baseline, with normal ambulation and spontaneous urination. Early treatment should be considered in conjunction with specialist consultation and a discussion of the risks and benefits with the patient or guardian. The patient’s workup was notable for a positive Monospot test, which may suggest a recent Epstein-Barr virus infection; however, EBV-specific serological testing would be required for confirmation. The etiologies of TM are diverse and include bacterial and viral infections, post-vaccination events, and neurological autoimmune disorders such as myelin oligodendrocyte glycoprotein antibody-associated disease and neuromyelitis optica spectrum disorder. Despite the extensive diagnostic workup performed in this case, evaluation for immune-mediated causes was not obtained at the time of presentation. Regardless of etiology, patients with TM may present with urinary retention and acute weakness that can progress relatively rapidly. Therefore, TM should remain in the differential diagnosis when these features are present. Although rapid progression of symptoms does not necessarily correlate with worse outcomes, it should not be used as an independent prognostic factor for symptom progression within the first 24 hours [4]. Early clinical suspicion is important to facilitate prompt evaluation and treatment, particularly given the potential for progression and significant neurological morbidity. The patient in this case met several clinical and investigative features supporting the diagnosis of TM, including urinary retention and lower-extremity weakness, MRI findings demonstrating T2 hyperintensity, and CSF pleocytosis. The diagnosis of TM requires exclusion of spinal cord compression and evidence of spinal cord inflammation. MRI and CSF findings can provide supportive evidence, as demonstrated in this case. Regarding the etiology, the patient’s TM may have been associated with a recent viral illness; however, a specific viral etiology was not definitively established. Magnetic resonance imaging is an important diagnostic tool in TM and may also provide prognostic information by demonstrating spinal cord lesions with T2 hyperintensity [1].

## Conclusions

Pediatric TM is an inflammatory disorder of the spinal cord that can result in significant neurological deficits. It has diverse etiologies and can occur in children. Pediatric patients generally have better outcomes than adults. Early recognition and diagnosis are crucial to facilitate timely treatment, including in the emergency department. The clinical presentation of TM can overlap with that of other neurological disorders. Therefore, clinicians should maintain a high index of suspicion and consider early initiation of corticosteroid therapy when TM is suspected, in accordance with current treatment recommendations. Given the potential for rapid progression of neurological deficits, the potential benefits and risks of treatment should be considered promptly in consultation with appropriate specialists. In this case, the patient responded well to steroids and IVIG and returned to his functional baseline without residual neurological deficits.
